# Innate-Like and Conventional T Cell Populations from Hemodialyzed and Kidney Transplanted Patients Are Equally Compromised

**DOI:** 10.1371/journal.pone.0105422

**Published:** 2014-08-21

**Authors:** Marine Baron, Renata Belo, Dominique Cathelin, Lucia Moreira-Teixeira, Claire Cartery, Eric Rondeau, Laurent Mesnard, Maria Leite-de-Moraes

**Affiliations:** 1 INEM (Institut Necker Enfants Malades), CNRS UMR 8253, INSERM UMR 1151, Université Paris Descartes, Sorbonne Paris Cité, France, Hôpital Necker Enfants Malades, Paris, France; 2 INSERM UMR S 702, Université Pierre et Marie Curie, Assistance Publique-Hôpitaux de Paris, Hôpital Tenon, Paris, France; 3 AP-HP, Hôpital Tenon, Urgences Néphrologiques & Transplantation Rénale, Universite Pierre et Marie Curie, Paris, France; 4 Nephrology and Dialysis, Tenon Hospital, Assistance Publique-Hôpitaux de Paris, Hôpital Tenon, Paris, France; Karolinska Institutet, Sweden

## Abstract

Clinicians are well aware of existing pharmacologically-induced immune deficient status in kidney-transplanted patients that will favor their susceptibility to bacterial or viral infections. Previous studies indicated that advanced Stage 4–5 Chronic Kidney Disease might also be regarded as an immune deficiency-like status as well, even though the mechanisms are not fully understood. Here, we analyzed the *ex vivo* frequency and the functional properties of both conventional and innate-like T (ILT) lymphocyte subsets in the peripheral blood of 35 patients on hemodialysis, 29 kidney transplanted patients and 38 healthy donors. We found that peripheral blood cell count of ILT cells, as iNKT (invariant Natural Killer T) and MAIT (mucosal-associated invariant T), were significantly decreased in hemodialyzed patients compared to healthy controls. This deficiency was also observed regarding conventional T cells, including the IL-17-producing CD4^+^ Th17 cells. Pertaining to regulatory T cells, we also noticed major modifications in the global frequency of CD4^+^CD25^+^Foxp3^+^ T lymphocytes, including the resting suppressive CD45RA^+^Foxp3^lo^ and activated suppressive CD45RA^−^Foxp3^hi^ T cell subpopulations. We found no significant differences between the immune status of hemodialyzed and kidney-transplanted subjects. In conclusion, we demonstrated that both ILT and conventional T cell numbers are equally impaired in hemodialyzed and kidney-transplanted patients.

## Introduction

Chronic kidney disease (CKD) is a common disease characterized by the progressive loss of the renal function that may lead to the initiation of treatment by replacement therapy such as hemodialysis or kidney transplantation (KT). Besides classical complication of chronic renal failure such as hypertension, fluid overload, hypocalcemia or even anemia, complications as a result of infections, especially caused by bacteria, are also an important source of morbidity and mortality in these patients, particularly at the terminal stage (CKD-5) [1]. A possible explanation for this susceptibility to infections is their frequent hospitalization, overuse of antibiotics and other confounding factors like diabetes or auto-immune related disease that are commonly associated with their kidney disease. Therefore, compromised immune responses may also explain this susceptibility to infectious agents [2]. Indeed, immunological abnormalities reported so far for CKD-5 patients include reduced phagocytic functions, defective antigen presentation and impaired B and T cell responses [3]–[8]. The mechanisms responsible for these deficiencies are not fully elucidated.

T lymphocytes are currently considered as major players to coordinate adaptive immune responses against infections. Recently, several studies highlighted the participation of a new group of T cells, called innate-like T (ILT) cells, which are on the frontier between innate and adaptive immune responses. Here, we focused our attention on two ILT populations, namely invariant Natural Killer T (iNKT) and mucosal-associated invariant T (MAIT) cells that display conserved significant homologies between mouse and human [9], [10], [11]. These lymphocytes produce a broad range of cytokines few minutes after stimulation allowing them to modulate both innate and acquired immunity in a large spectrum of inflammatory diseases [9], [10], [11], [12]. They express a highly restricted T cell receptor (TCR) repertoire composed in humans of a single invariant V**α**24J**α**18 and V**α**7.2J**α**33 for iNKT and MAIT cells, respectively. In contrast to conventional T cells that recognize peptides, iNKT cells recognize glycolipids presented by CD1d while MAIT cells are activated by vitamin B metabolites presented by the MHC-related protein 1 (MR1) molecules [13], [14]. Both iNKT and MAIT cells are generally regarded as protective against infections and we reported that iNKT cells also attenuated the development of anti-glomerular basement membrane glomerulonephritis in a murine model [15], [16], [17], [18]. Herein, we analyzed the frequency of these innate-like T lymphocytes, namely iNKT and MAIT cells, and of conventional T cells in the peripheral blood of CKD-5/Hemodialyzed (HD) and kidney transplanted patients.

## Materials and Methods

### Patients

Patient demographic characteristics and most relevant clinical data are shown in Table 1. All patients entered in this study after providing their informed consent. The study was conducted according to the procedures of the Declaration of Helsinki and to local ethic committee rules (Commission de Protection des Personnes, Ile de France). Patient's anonymity was protected. Thirty-seven HD patients, 31 kidney transplanted patients and 38 healthy volunteer's donors, considered here as controls, (Table 1) (obtained after signed informed consent managed by the French Blood Department) participated in the study.

**Table 1:** Patient demographic characteristics.

Characteristic	Value
**Non-transplanted patient**	
N	35
Age (yr; mean ± SEM)	40.4±1.74
Gender (female/male)	13/22
Cause of ESRD:	
Vascular nephropathy (hypertensive sclerosis, thrombotic microangiopathy)	8
Autoimmune related nephropathy (systemic lupus erythematosus, Wegener granulomatosis, rheumatoid purpura, idiopathic GEM)	8
Primary glomerulonephritis (focal segmental glomerulosclerosis, IgA nephropathy, Alport syndrome)	7
Secondary glomerulonephritis (diabetes mellitus, type 1 diabetes, sickle cell disease)	5
Tubulointerstitial nephritis (reflux nephropathy, myeloma)	4
Congenital/hereditary nephropathy (polycystic kidney)	4
Unknown	1
Blood lymphocyte counts per mm^3^ (mean ± SEM)	1335±187
**Transplanted patient**	
N	29
Age (yr; mean ± SEM)	40.1±1.83
Gender (female/male)	10/19
Years post-transplantation (mean ± SEM)	3.34±0.59
Cause of ESRD:	
Primary glomerulonephritis (Alport syndrome, IgA nephropathy, focal segmental glomerulosclerosis)	7
Congenital/hereditary nephropathy (polycystic kidney, Fabry disease, tuberous sclerosis, Bor syndrome)	6
Unknown	6
Tubulointerstitial nephritis (reflux nephropathy)	5
Vascular nephropathy (hypertensive sclerosis, atypical hemolytic uremic syndrome)	4
Autoimmune related nephropathy (systemic lupus erythematosus)	1
Blood lymphocyte counts per mm^3^ (mean ± SEM)	1316±147
MDRD (mL/min; mean ± SEM)	61.9±4.03
**Healthy donors**	
N	38
Age (yr; mean ± SEM)	30.7±2.2
Gender (female/male)	15/23
Blood lymphocyte counts per mm^3^ (mean ± SEM)	1794±213

Two hemodialysed patients had glomerulonephritis and vascular nephropathy at the same time.

### Cell preparation

Blood samples were obtained from CKD or kidney transplanted patients (End Stage Renal Disease) from Tenon Hospital (Kidney Emergencies and Renal Transplantation and nephrology and hemodialysis units), Paris, France. All analyses were performed on freshly isolated peripheral blood mononuclear cells (PBMC) from 10 to 20 mL of blood by density-gradient centrifugation (Ficoll-Paque PLUS; GE Healthcare). Cell-surface staining was performed in PBS buffer containing 2% FCS and 0.01% NaN_3_ on ice, as previously described [12], [19]. Cells were first stained with PBS57-loaded or empty-CD1d-tetramers (National Institutes of Health Tetramer Core Facility), then with the following directly conjugated monoclonal antibodies (eBioscience): anti-CD3, anti-CD4, anti-CD8, anti-CD25, anti-CD45RA, anti-CD161, anti-TCRV**α**7.2 and/or anti-Foxp3. Intra-cellular analysis of Foxp3 was performed after fixation and permeabilization using Foxp3 staining buffers (eBioscience). Data were acquired on a FACSCanto II flow cytometer (BD Biosciences) with the use of FACSDiva Version 6.1.3 software (BD Biosciences) and were analyzed with the FlowJo Version 8.5.3 software (TreeStar). Lymphocyte subpopulations were analyzed within the lymphocyte gate on forward and side-scatter plots. Results were expressed in absolute numbers per mm^3^ of peripheral blood.

### Intracellular cytokine staining

For intracellular cytokine staining, PBMC were incubated for five hours with PMA (25 ng/mL), Ionomycin (1** µ**g/mL) and Brefeldin A (10** µ**g/mL, all from Sigma-Aldrich). Cells were stained with CD1d-tetramer, washed and fixed with 4% paraformaldehyde and permeabilized with 0.5% saponin (Sigma-Aldrich) before further incubated with anti-CD3, anti-CD4, anti-CD8, anti-IL-4, anti- IFN**γ** anti-IL-17 antibodies. Isotype-matched antibodies were used to define marker settings for cytokines antibodies.

### Statistical Analysis

Statistical analyses were performed using GraphPad Prism (version 6.0 for Mac OS X, San Diego, California USA). Groups were compared using the nonparametric Mann-Whitney U test for non-normally distributed variables. All p values were two-tailed, and the statistical significance level was defined as a p<0.05.

## Results and Discussion

Clinical data are presented at Table 1. In sum, of the 35 HD patients, 13 were female and 22 male, with an age of 40.4±1.6 (range 19-59) years at the time of blood collection. The gender and age of healthy donors were 15 females/23 males, 30.7±2.2 years old (range 20–64). The cause of ESRD (End Stage Renal Disease) for these HD patients was vascular nephropathy (8 patients), autoimmune related nephropathy (8 patients), primary glomerulonephritis (7 patients), secondary glomerulonephritis (5 patients), tubulointerstitial nephritis (4 patients), congenital/hereditary nephropathy (4 patients) or unknown (1 patient). Two HD patients had both glomerulonephritis and vascular nephropathy. Eleven HD patients received corticoids associated or not with immunossuppressive drugs (calcineurin inhibitors or selective inhibitors of inosine monophosphate dehydrogenase or antimetabolite-drug). All analysis performed in our study were made including or not these patients. Statistic differences reported below were obtained whatever immunosuppressed patients were excluded or not (data not shown). For this reason, they were maintained in the study.

Among the 31 kidney-transplanted patients, 11 were female and 20 male, with an age of 39.7±1.7 (range 20–59) years at the time of blood collection. Patients were analyzed about 3.34±0.59 years post-transplantation. The cause of ESRD for these KD patients was primary glomerulonephritis (7 patients), congenital/hereditary nephropathy (6 patients), unknown (6 patient), tubulointerstitial nephritis (5 patients), vascular nephropathy (4 patients), autoimmune related nephropathy (2 patients) or secondary glomerulonephritis (1 patient). Their MDRD was 62.1±3.78 mL/min.

**Figure 1:**
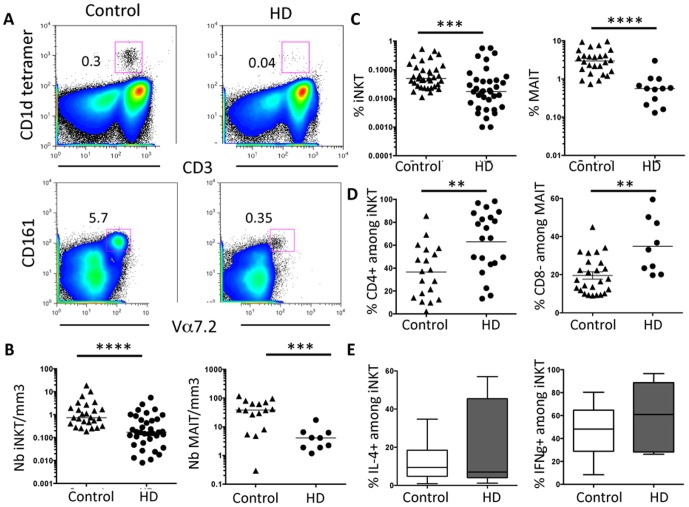
iNKT cell deficit in HD patients. (A) iNKT cells were double stained by anti-CD3 and the PBS57-loaded CD1d-tetramer. Representative FACS profile showing the percentage of iNKT and MAIT cells in control versus HD patients. (B and C) HD patients had significant low numbers (B) and percentage (C) of peripheral blood iNKT and MAIT cells when compared to healthy donors. Bars represent the median. (D) The frequency of CD4^+^ and CD8^−^ subsets respectively among gated iNKT and MAIT cells in control versus HD patients is represented. (E) The percentage of IL-4^+^ or IFN**γ** ^+^ cells among gated iNKT lymphocytes was assessed after 5 h stimulation with PMA and ionomycin. Box-and-whisker plots are used to represent the distributions. The bottom and the top of a box represent the 5th and 95th percentiles, and the bar in the box shows the median. **, P<0.001; ***, P<0.0005; ****, P<0.0001 versus controls.

Herein, we analyzed the frequency of iNKT and MAIT cells in the peripheral blood of CKD-5/Hemodialyzed (HD) patients. iNKT cells were identified by CD1d/PBS57 tetramers and MAIT cells by the expression of CD3, CD161 and TCR V**α**7.2 chain (Figure 1A). We found that the absolute number of iNKT and MAIT cells was drastically reduced in HD patients compared to control healthy donors (Figure 1B). We confirmed previous reports showing that the number of total CD3^+^ T cells was diminished in these patients (808.2±130/mm^3^ in HD patients versus 1296±156/mm^3^ in health donors) [20]. iNKT and MAIT cell deficiency was also observed in terms of percentage among gated CD3^+^ T cells (Figure 1C), thus confirming their pronounced deficiency in HD patients. In addition, the subset distribution of these particular T cell populations was also committed since we found that the percentage of CD4^+^ and CD8^−^ subsets were enhanced among gated iNKT and MAIT cells, respectively (Figure 1D) compared to controls. The reduced number of these distinct iNKT or MAIT subsets limited a more accurate analysis of their specific cytokine profile. However, *ex vivo* analysis of cytokine-producing capacities did not outline significant differences in the percentage of IL-4^+^ or IFN**γ** ^+^ among gated iNKT cells between patients and healthy controls (Figure 1E) indicating that these cells are functional, but yet in very few numbers in HD patients.

**Figure 2:**
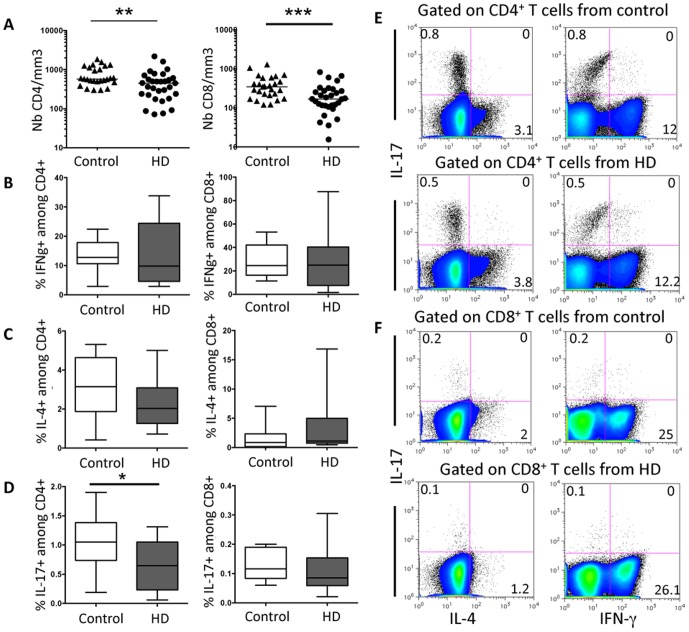
CD4^+^ and CD8^+^ T cell numbers are reduced in HD patients. (A) CD4^+^ and CD8^+^T cell numbers are significant decreased in the peripheral blood of HD patients compared to healthy donors. Bars represent the median. (B) The percentage of IFN**γ** ^+^ cells among gated CD8^+^ or CD4^+^ T lymphocytes or (C) the percentage of IL-4^+^ and IL-17^+^ cells among gated CD4^+^ T lymphocytes was assessed after 6 h stimulation with PMA and ionomycin. Box-and-whisker plots are used to represent the distributions. The bottom and the top of a box represent the 5th and 95th percentiles, and the bar in the box shows the median. *, P<0.01; **, P<0.001; ***, P<0.0005 versus controls. Representative FACS analysis of IL-4, IL-17 and IFN**γ** production by gated CD4^+^ (E) or CD8^+^ (F) T cells from health donor controls (top) or HD patients (bottom) are represented.

Deficiency in peripheral blood cell count and percentage was also observed in HD patients concerning CD4^+^ and CD8^+^ T cells (Figure 2A). Using a short, 6 hours, polyclonal stimulation, we found no significant difference in the ability of *ex vivo* CD4^+^ T cells to secrete IFN**γ** (Figure 2B and 2E). Similarly, the percentage of IFN**γ**-producing CD8^+^ T cells was unchanged in HD patients compared to controls (Figure 2B and 2F). This was also true regarding IL-4-producing CD4^+^ (Figure 2C and 2E) or CD8^+^ T cells (Figure 2C and 2F). Conversely, the frequency of IL-17A (or IL-17)-producing CD4^+^ T cells was significantly reduced in HD patients (Figure 2D and 2E). No significant difference was observed concerning the ability of CD8+ T cells from HD patients to secrete IL-17 (Figure 2D and 2F). IL-17-producing Th17 CD4^+^ T cells are usually considered as playing an important role against infections including fungal infections [21]. Polymorphisms within the IL-17E and IL-17RA genes are associated with end stage renal disease [22], suggesting that IL-17 family or its receptors could influence the severity of the kidney inflammatory response. Together, our findings reveal that CD4^+^ T cells in HD patients are impaired in number with a pronounced deficiency in their IL-17-producing subset, namely CD4^+^ Th17 cells.

**Figure 3:**
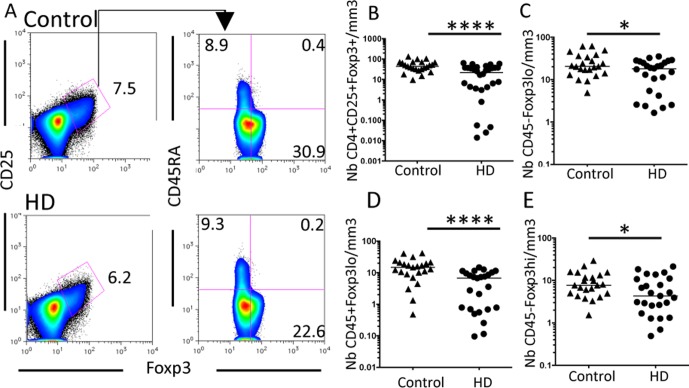
Global Foxp3^+^ T cell numbers are reduced in HD patients. (A) Representative FACS analysis of CD4^+^CD25^+^Foxp3^+^T cells and their distinct subsets on PBMC from control (top) and HD patients (bottom). (B) CD4^+^CD25^+^Foxp3^+^T cell numbers are reduced in HD patients. (C to E) Among these gated cells, cytokine-secreting CD45RA^−^Foxp3^lo^ nonsuppressive (C), resting suppressive CD45RA^+^Foxp3^lo^ (D) and activated suppressive CD45RA^−^Foxp3^hi^ (E) cells, all these subsets are reduced in HD patients compared to controls. Bars represent the median. *, P<0.01; ****, P<0.0001.

In addition to the analysis of *ex vivo* cytokine-producing capacities, we also focused our attention on regulatory Foxp3^+^CD4^+^ T (Treg) cells that play a key role in the regulation of inflammatory responses. Treg cells are usually designed as CD25^high^Foxp3^+^ cells. We found that HD patients presented a global deficiency in the CD4^+^Foxp3^+^CD25^+^ T cells count (Figure 3A and 3B). Previous reports have highlighted that TCR activated human T cells can express Foxp3 and CD25 but these cells are not suppressive [23], [24]. To address this issue, new combinations of markers are now used to distinguish suppressive from nonsuppressive Foxp3^+^ T cells. A pertinent example is the analysis of CD45RA expression associated with distinct levels of Foxp3 [25] (Figure 3A). This approach revealed that the peripheral number of cytokine-secreting CD45RA^−^Foxp3^lo^ nonsuppressive (Figure 3C), resting suppressive CD45RA^+^Foxp3^lo^ (Figure 3D) and activated suppressive CD45RA^−^Foxp3^hi^ (Figure 3E) T cell subpopulation were significantly reduced in HD patients compared to controls. Globally these findings endorse the above results showing that the immune system of HD patients is dysregulated not only at the level of non conventional ILT iNKT and MAIT cells but also of conventional effector and regulatory T cells. This global immune dysregulation might favor the susceptibility of these patients to infections.

**Figure 4:**
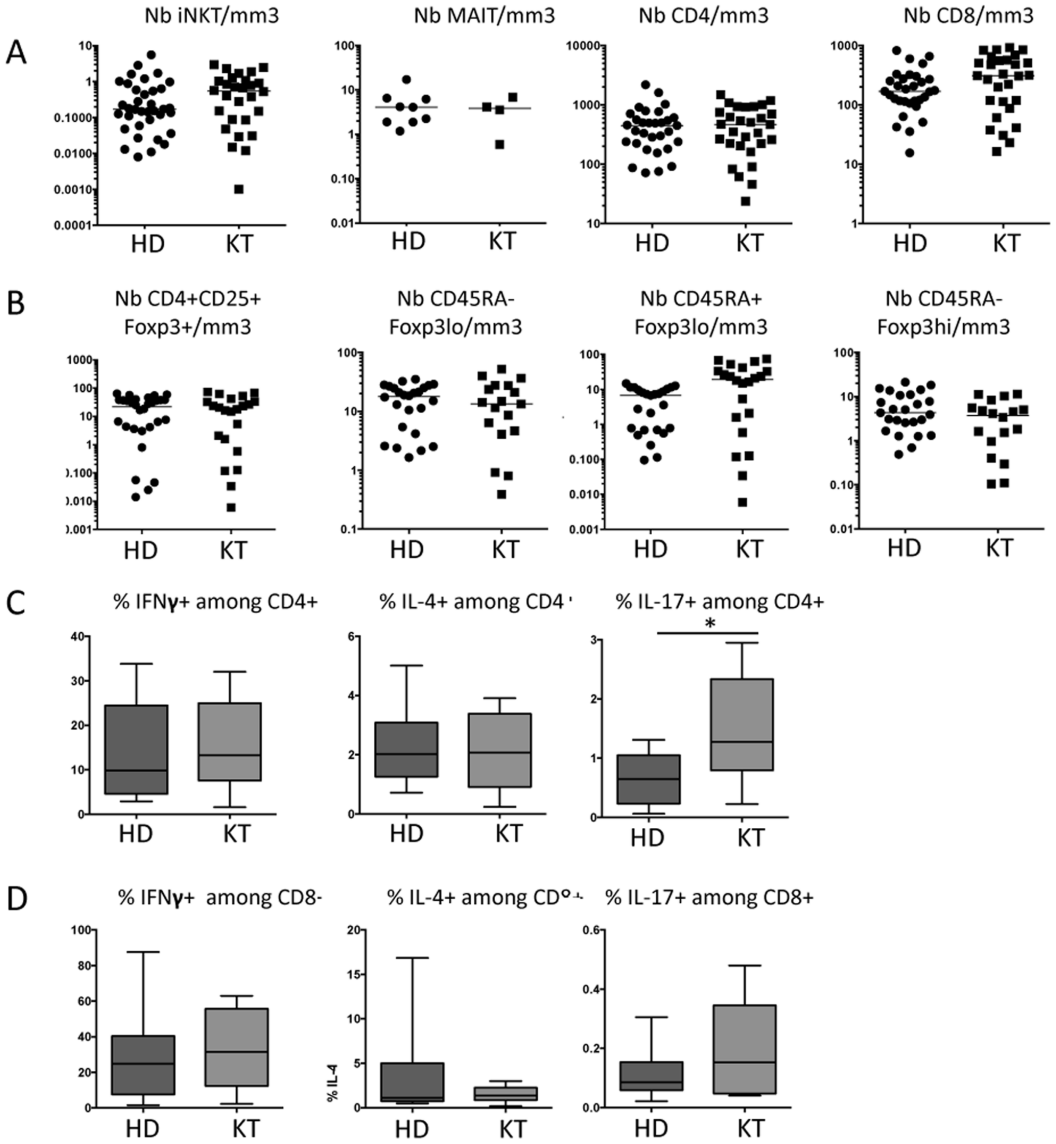
Both ILT and conventional T cells are equally deficient in HD and KD patients. (A) HD patients had no significant differences in their number of iNKT, MAIT, CD4^+^ and CD8^+^ T cells when compared to kidney transplanted (KT) patients. Bars represent the median. (B) CD4^+^CD25^+^Foxp3^+^T cell numbers are similarly reduced in HD as in KD patients. Among these gated cells, cytokine-secreting CD45RA^−^Foxp3^lo^ nonsuppressive, resting suppressive CD45RA^+^Foxp3^lo^ and activated suppressive CD45RA^−^Foxp3^hi^ cell levels, are also similar in HD and KD patients. (C and D) The percentage of IFN**γ** ^+^, IL-4^+^ or IL-17^+^ cells among gated CD4^+^ (C) or CD8^+^ (D) T lymphocytes was assessed after 6 h stimulation with PMA and ionomycin. Box-and-whisker plots are used to represent the distributions. The bottom and the top of a box represent the 5th and 95th percentiles, and the bar in the box shows the median. (A and B) Bars represent the median. *, P<0.01.

These results aimed us to ask a key question: are these immune deficits observed in HD patients similar to those reported in kidney-transplanted persons? Kidney transplantation is a common treatment for HD patients. We confirmed an immune deficit in kidney-transplanted patients in terms of T cell counts when compared to healthy donors (Table 2). Interestingly, the analysis of kidney-transplanted patients clearly shows that their immune system is equally impaired as those from ERDS patients since we found no significant difference between the distinct ILT and conventional T cell populations tested (Figure 4). Indeed, the number of peripheral blood iNKT, MAIT, CD4^+^, CD8^+^ and CD4^+^CD25^+^Foxp3^+^ T cell populations, including CD45RA^−^Foxp3^lo^ nonsuppressive, resting suppressive CD45RA^+^Foxp3^lo^ T cell and activated suppressive CD45RA^−^Foxp3^hi^ T cell subpopulations were similar in kidney transplanted and HD patients (Figure 4A and 4B). Concerning their cytokine profile, the frequency of CD4- or CD8-producing IFN**γ** or IL-4 was similar between HD and KT patients (Figure 4C and 4D). IL-17-producing T cells was slightly different since the percentage of IL-17^+^ among gated CD4^s^ T cells was enhanced in KT compared to HD patients (Figure 4C). However, this was not confirmed in terms of total cell counts of IL-17-producing CD4^+^ T cells (5.9±2.6 versus 4.3±1.2×10^3^/ml for HD versus KT patients, respectively).

**Table 2:** Numbers of distinct T cell populations in the peripheral blood of kidney-transplanted versus healthy donors.

T cell populations	Number per mm3 in		p
	transplanted patients	healthy donors	
iNKT	0.76±0.15	2.31±0.78	0.0360
MAIT	3.78±1.28	44.76±8.50	0.0107
CD4	501.4±70.4	765.7±85.1	0.0098
CD8	365.3±51.7	407.0±53.9	0.4875
CD4+Foxp3+CD25+	22.46±4.87	51.81±6.43	0.0005
CD45RA-Foxp3lo	16.26±3.45	25.94±3.31	0.0394
CD45RA+Foxp3lo	7.12±1.65	15.78±2.27	0.0036
CD45RA-Foxp3hi	4.20±0.90	9.71±1.36	0.0017

Results are expressed as mean ± SEM of cell counts in the peripheral blood of kidney-transplanted versus healthy donors. Mann Whitney test was used to compare the sample.

p<0.05 are considered as significant.

Overall, our findings clearly show that HD patients presented similar immune dysfunctions as transplanted patients. This information is of high clinical value, because clinicians are well aware of the poor immunological status of transplanted patients but underestimate the immunological status of HD patient *per se*. Furthermore, our findings concerning ILT cells, namely MAIT and iNKT cells, are the first evidence that these lymphocytes are reduced in the peripheral blood of HD and kidney-transplanted patients. Their deficiency likely favors the susceptibility of these patients to infections. T cell counts were also reduced in terms of conventional T cell subsets namely CD8^+^, CD4^+^, Th17, CD4^+^CD25^+^Foxp3^+^ and their subsets: nonsuppressive CD45RA^−^Foxp3^lo^, resting suppressive CD45RA^+^Foxp3^lo^ T cell and activated suppressive CD45RA^−^Foxp3^hi^ T cell subpopulations. The low number of resting suppressive CD45RA^+^Foxp3^lo^ and activated suppressive CD45RA^−^Foxp3^hi^ T cell subsets, could contribute to the global dysregulation of the immune systems observed so far in CKD and transplanted patients.

In conclusion, kidney failure results in a global T cells immune dysfunction that can contribute to enhance the risk of complications due to infections in particular. Our, data reinforce the underestimated immune dysfunction found in HD patients, which appears similar to kidney-transplanted patients. Overall our findings emphasize the importance of tight clinical fellow-up of hemodialyzed patients, especially in an infectious context.
